# Inheritance of the acoustic signal parameters in interspecific hybrids of the bank (*Myodes glareolus*) and the Tien Shan (*M. centralis*) voles

**DOI:** 10.1186/s12862-019-1374-7

**Published:** 2019-02-26

**Authors:** Marina Vladimirovna Rutovskaya

**Affiliations:** 0000 0001 2192 9124 grid.4886.2 A.N. Severtsov Institute of Ecology and Evolution, Russian Academy of Sciences, Moscow, Russia

**Keywords:** Sound communication, Rodents, Inheritance of vocal signals

## Abstract

**Background:**

The continuity of behavioral responses in the traits of offspring can be interpreted ambiguously because animal behavior can be transmitted from generation to generation genetically or can be trained. Inheritance of the sound signals characteristics in the absence of directional selection is of particular interest, and that was the purpose of the present work.

**Results:**

Comparisons of distress signals of hybrids of the Tien Shan vole females *Myodes centralis* (Kyrgyzstan) and the bank vole males *M. glareolus suecicus* (the Tver region), and the parent species were made. Acoustic signals of the hybrids and parent species were compared using the variance and discriminant analysis. The distress signals of the Tien Shan voles were shorter, had lower peak frequency than the signals of the bank voles. The peak frequency of hybrids signals was closer to that of the bank voles. However, the duration of the hybrids sounds, on the contrary, was closer to that of the Tien Shan voles. The expression of the noise component in the sounds of the hybrids occupied an intermediate position. The discriminant analysis of distress signals in the Tien Shan and bank voles showed 94% of correct attributions.

**Conclusions:**

Our results confirm that the acoustic signals parameters were inheritance intermediate. The paternal genome (the bank voles) had greater influence on parameters of the sound signals in hybrids.

## Background

In a stress situation, the red-backed voles emit acoustic signals (distress signals) that reflect their emotional state [1, 2]. These signals are typical for many rodents, but the physical parameters of these sounds vary in different species [3–5]. This is the case for both the bank and Tien Shan voles [1]. A number of authors have pointed the genetic inheritance of acoustic signal parameters in mammals, for example in the common voles *Microtus arvalis* [6] and the bank voles *Myodes glareolus* [7]. The acoustic signal parameters are inherited by hybrids intermediately, which was shown, for example, for ground squirrels [8], hoofed mammals [9], and pikas [10]. The inheritance of morphological characters of a species correlates with that of acoustic signal parameters [7, 11].

The goal of this study was to determine the regularities of the inheritance of acoustic signal parameters by the hybrids of the bank and Tien Shan voles.

## Materials and methods

The work was conducted at the A.N. Severtsov Institute of Ecology and Evolution, the Joint Usage Center’s “Collection of live mammals” at the Chernogolovka biological station. The hybrids of the bank vole male (*Myodes glareolus suecicus*) from Tver region and the Tien Shan vole female (*M. centralis*) from the Kyrgyzstan were obtained. The parent species were represented by descendants of the second - to third vole generations. We recorded four hybrid females, five males and five females of the Tien Shan voles and two females and three males of the bank voles. The distress signals were recorded in laboratory conditions, during handling, using a Tascam NoDA-P1 professional digital tape recorder (United States) and SENNHEISER K6 microphone (Germany). During handling, we removed the animal from the living cage and held it by the scruff for 10 min. 10–20 distress signals were measured from each animal in a personal computer using the AviSoft SASLab pro professional program (version 4.2). The call duration was measured onscreen using the standard marker cursor in the spectrogram window (Hamming window, FFT 512 points, frame 100%, and overlap 87.5%) that provided 1.45 ms time resolution and 43 Hz frequency resolution. For each vocalization, the following parameters were measured in automatic mode: maximum amplitude frequency (peak frequency), medium quartile, and entropy. The maximum value of the fundamental frequency (hereafter, fundamental frequency ˗ the lowest frequency of a harmonic signal produced by the vocal cord oscillation) was measured using the harmonic cursor from the harmonic power spectrum (logarithmic) at the maximum value of the modulated signal. All the measured signals were divided by structure into harmonic, noise and mixed. If it was impossible to distinguish a harmonic relation between the component frequencies at “harmonic power spectrum” window, the signal was classified as noise calls. The fundamental frequency of the noise calls was not measured. The mixed signals included the fundamental frequency and broadband noise over it.

The lower, medium, and upper quartiles of the power spectrum are the frequencies, below which there are 25, 50, and 75% of all energy of the spectrum, respectively. The parameter “entropy” that is used for the quantitative estimate of a tone, i.e., the ratio between the noise and harmonic energy in the power spectrum, was calculated as the ratio of the geometric mean to the arithmetic mean of the spectrum.

The obtained results were processed using the Statistica Ultimate Academic v. 12 program for Windows (StatSoft, Russia). Acoustic parameters of the hybrids and parent calls, that had normal distribution, were compared using the variance and discriminant analysis in combination with cross-validation (check of the reliability of discrimination keys), and the obtained proportions were compared by the correct attribution by the criterion χ^2^ with a random value that was obtained as a result of the randomization procedure [12]. The contribution of different sound parameters to discrimination was determined according to the value of the Wilks lambda. To perform the cross-validation procedure, each sample was divided in half randomly; then, one part of the sample was used to calculate recognition keys that were tested based on the second half of the sample. Peak frequency had not normal distribution, so it was compared using nonparametric Kruskal-Wallis ANOVA, median test.

During this work, I adhered to the ‘Guidelines for the treatment of animals in behavioral research and teaching’, published by the Animal Behavior Society [13], and to the laws on animal welfare for scientific research of the Russian Federation, where the study was conducted. A.N. Severtsov Institute of Ecology and Evolution provided full approval for this purely observational research.

## Results

Distress signals of the Tien Shan voles were shorter compared with those of the bank voles and had a higher fundamental frequency and less noise components. The peak frequency of the Tien Shan vole’s sounds was lower compared with that of the bank voles (Table 1).

**Table 1:** Parameters of the female’s distress signals of the bank vole *Myodes glareolus* and the Tien Shan voles *M. centralis* and their hybrids, and comparison by the single-factor ANOVA method

Parameters	The Tien Shan voles	Hybrids	The bank voles	ANOVA
	*n* = 118	*n* = 108	*n* = 78	
Duration, s	0.027 ± 0.001^a^	0.023 ± 0.001^b^	0.054 ± 0.004^c^	**F**_**2,301**_ **= 182.2** ***p*** **= 0.001**
Fundamental frequency, kHz	3.8 ± 0.1^a^	3.2 ± 0.1^b^	1.5 ± 0.1^c^	**F**_**2,128**_ **= 23.45** ***p*** **= 0.001**
Modulation range, kHz	2.5 ± 0.1^a^	1.6 ± 0.1^b^	0.6 ± 0.1^c^	**F**_**2,128**_ **= 45.35** ***p*** **= 0.001**
Quartile 50%, kHz	4.0 ± 0.1^a^	7.9 ± 0.1^b^	6.8 ± 0.1^c^	**F**_**2,301**_ **= 81.6** ***p*** **= 0.001**
Entropy	0.520 ± 0.005^a^	0.586 ± 0.008^b^	0.637 ± 0.004^c^	**F**_**2,301**_ **= 200.8** ***p*** **= 0.001**

Significant values are highlighted in bold. The different letter-indices in the line denote significantly different values, by using Tukey Post-hock analysis

**Fig. 1:**
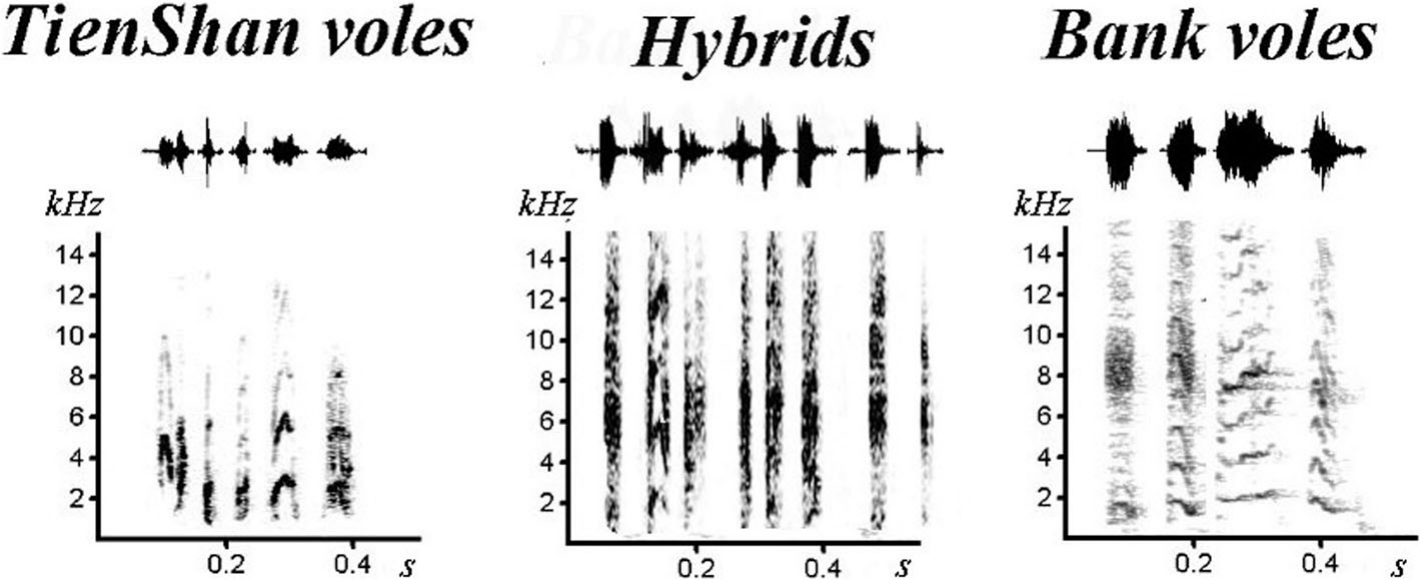
Examples of sonograms and oscillograms of female’s distress signals of the bank and Tien Shan voles and their hybrids

There were 3–10 harmonic and a noise component included at the vole’s signals (Fig. 1). The fundamental frequency was about 1.5 kHz in the bank voles and more than 3 kHz in the Tien Shan vole’s sounds. The modulation of the fundament frequency had a ∩ − shaped form. The modulation range of the fundament frequency was different in all species: it was higher in signals of the Tien Shan voles. The peak frequency is high in the bank vole’s signals (median – 7.6 kHz) and low in the Tien Shan vole’s signals (median – 2.8 kHz), that differed significantly (χ^2^ = 57.22, *p* = 0.001). The noise component was more pronounced in the bank vole’s sounds, which was reflected in the entropy value.

The hybrid’s signals differed significantly from the sounds emitted by parent species, except for the peak frequency of the signal, which was close in hybrids (median – 7.6 kHz) and in the bank voles (Multiple comparisons z = 0.0829, *p* = 1.0). The discriminant analysis based on the three parameters of distress signals (duration, quartile 50% and entropy) of the bank and Tien Shan voles showed 94.4% of correct attributions, which was very close to the value that was obtained by cross-validation (92.9%, χ^2^ = 0.34, *p* = 0.56), but surpassed the random value that was obtained by randomization (52.88 ± 0.4%, *n* = 100, χ^2^ = 69.44, *p* = 0.001). All parameters contributed to the differences between signals of the studied vole species.

**Fig. 2:**
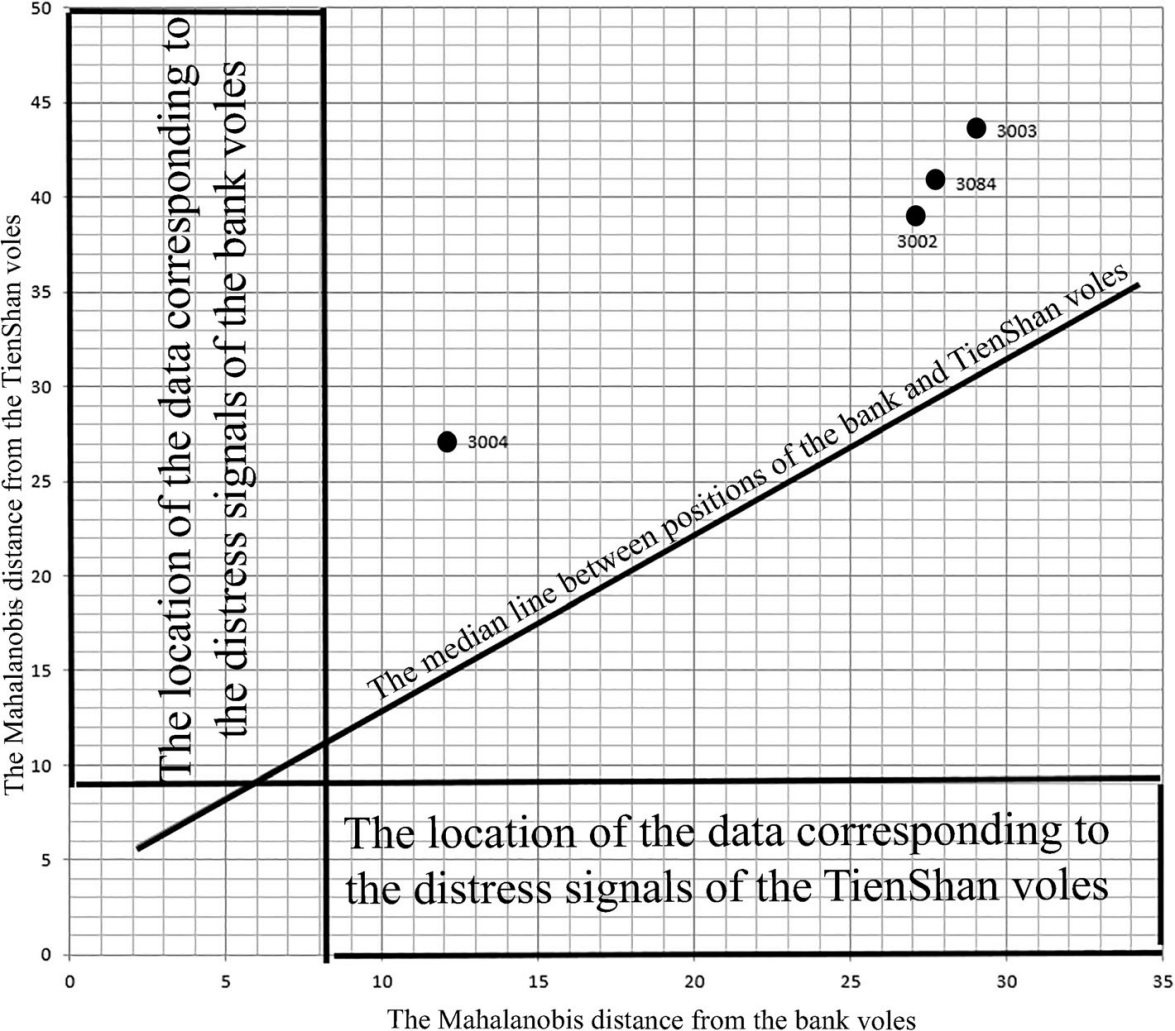
The location of hybrids according to the parameters of distress signals in the axes of the Mahalanobis distances between the parent species

The value of the squared Mahalanobis distances for each individual from the parent species was calculated based on the three signal parameters (duration, quartile 50% and entropy) (Fig. 2), the distress signals of hybrids differ from those of the parental species, and generally occupied an intermediate position in the Mahalanobis coordinate system, but closer to the bank voles. Therefore, some influence of the paternal parent species was observed on the formation of frequency characteristics of sounds in the hybrids, which have the same peak frequency of the distress signals as those of the bank voles.

## Discussion

The bank and Tien Shan voles are not sympatric species. The bank voles are widespread in European broad-leaved, coniferous-broad-leaved and dark coniferous forests, in the mountains and plains of Europe, Western Siberia and in the mountainous regions of Central Siberia, and the Tien Shan voles occupy an isolated area in the forest zone of the Tien Shan Mountains [14]. In nature, these species are not found in sympatry, unlike the widely overlapping areas of the red and bank voles, whose hybrids were obtained in captivity [15], and also have been found in nature [16]. Differences in the signals emitted by the red and bank voles, as well as by the bank and Tien Shan voles, was only of a statistical nature. In addition, the minimum and maximum values of the distress signals parameters in the Tien Shan and bank voles overlapped, since they had a large variability depending on the emotional state of individuals [1]. Also, there is the geographical variability of distress signals in the red and bank vole, for example, we compared two subspecies *M. g. suecicus* and *M. g. saianicus*, which had some statistical differences in frequency parameters, namely peak frequency and entropy, and two subspecies of the red vole *M. r. rossicus* и *M. r. rutilus*, which differ among themselves in almost all parameters, including duration and frequency, except for the entropy of sounds [17]. However, even taking into account the geographical variability of the signals of the bank and red vole, distress signals differ significantly among species in all parameters studied, and some regularities could be observed in its inheritance. So it was noticed that the hybrids of the red and bank voles produced sounds that had intermediate characteristics between the parent species, but the paternal influence was somewhat higher [7]. The same was noted for the bank and Tien Shan voles. Parental species differ in all parameters of distress signals, even taking into account the geographical variability of the bank vole [17]. According to the set of parameters the hybrid’s sound signals had intermediate position between the parental species, however, the influence of the paternal species (the bank voles) was stronger. Since we assume that these signals had no functional role and therefore were not affected by directed selection, the species specificity of signals was an indirect result of evolution of these species. [18] proved that the geographical variability of the advertisement song of a singing mouse (*Scotinomys teguina* and *S. xerampelinus*) was a result of gene drift, but not an adaptation to habitat conditions. Thus, geographic variability and the species specificity of the vocal repertoire can be either the result of directed selection, or may be a side effect of the species evolution that not related with the communication process. It may be gene drift or the morphological and physiological adaptations of the species that influenced the parameters of emitted sounds, including the influence of stress which can cause destabilizing selection [19, 20], increasing parameters variability. Nevertheless, we note that the revealed regularity has a systemic nature.

## Conclusions

Our results confirm that the acoustic signals parameters were inheritance intermediate. The paternal genome (the bank voles) had greater influence on parameters of the sound signals in hybrids.
